# Senior investigator biocommentary: Donna Ferriero

**DOI:** 10.1038/s41390-023-02892-9

**Published:** 2023-11-11

**Authors:** Donna Ferriero

**Affiliations:** Departments of Neurology and Pediatrics, University of California San Francisco, San Francisco, CA USA

I am a Distinguished Professor in the Departments of Neurology and Pediatrics at the University of California San Francisco Weill Institute for Neurosciences. My research involves investigating how injury to certain groups of neural cells affects the developing nervous system.

I got an MS in immunology at Rutgers University. I then took a job as a research assistant at the Roche Institute of Molecular Biology with a wonderful man, Frank Margolis, who studied olfaction. I then decided to get an MD and I was fortunate enough to get into the University of California San Francisco where I eventually did my residency with Dr Bruce Berg who got me excited about child neurology.
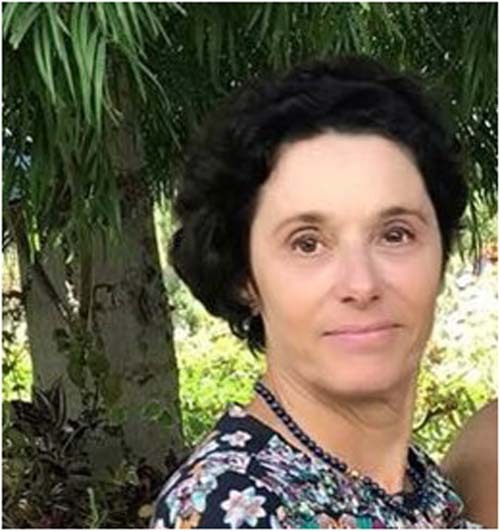


My interests in neonatal brain injury really developed in two ways—clinically and in the lab. During my training, I realized there was a big hole in our care of babies with brain injury. I used to be called to the NICU to pronounce babies brain dead without any participation in their care. I realized we needed to develop a specialized unit for brain care in the intensive care nursery. We were the first to found what we called the NICN, neurologic intensive care nursery, where we essentially mandated that an interdisciplinary team should comanage the patients, round together and make joint decisions about their care.

These early experiences also helped me to recognize that we needed to have a richer understanding of brain injury. When I took a position as Assistant Professor, I was introduced to Roger Simon who had a model of adult hypoxia ischemia. Together, we developed a neonatal model that later became the foundation for all of my research. We were the first to show that the deep gray matter of the brain is selectively vulnerable following maturation-dependent injury and we showed that the reason for that was an overabundance of excitatory receptors in that region which contributed to injury and cell death.

I also participated, along with a number of investigators including Allistair Gunn, in the CoolCap trial, which was the first trial of cooling the newborn brain using a cap that provided selective cooling only to the brain, which is now standard of care. Today in my lab, we are trying to figure out if we can identify metabolic profiles after therapeutic hypothermia that tell us which brains are still at risk and need further therapy after therapeutic hypothermia.

My advice to the next generation of pediatricians is to follow your passion. Take your time and explore what really drives you. Of course, I have had distractions along the way. Like many women, I have had children during my career and trying to find the perfect work–life balance and give your children the love and attention they need is critical. But I found that academia actually provided more flexibility than I might have had in private practice. The next generation of pediatric researchers gives me a lot to feel optimistic about. I just find them to be so smart and thoughtful and curious. It is a constant boost.

